# Supplementary material for: Resolving the full spectrum of human genome variation using Linked-Reads
Source: Genome Res. 2019 Apr;29(4):635–45. doi: 10.1101/gr.234443.118 (PMC6442396; doi:10.1101/gr.234443.118)
Supplement: Supplemental Material [file supp_gr.234443.118_Supplemental_longranger-2.2.zip › longranger-2.2.tar.bz2!/lr2.2-public/lib/stan/cmdstan/stan/doc/api/html/classstan_1_1mcmc_1_1stepsize__var__adapter-members.html]

Stan: Member List


|  |  |
| --- | --- |
|  | Stan  2.5.0  probability, sampling & optimization |


- Main Page
- Related Pages
- Namespaces
- Classes
- Files

- Class List
- Class Index
- Class Hierarchy
- Class Members

All Classes Namespaces Files Functions Variables Typedefs Enumerations Enumerator Friends Macros Pages

- stan
- mcmc
- stepsize\_var\_adapter

stan::mcmc::stepsize\_var\_adapter Member List

This is the complete list of members for stan::mcmc::stepsize\_var\_adapter, including all inherited members.

|  |  |  |
| --- | --- | --- |
| adapt\_flag\_ | stan::mcmc::base\_adapter | protected |
| adapting() | stan::mcmc::base\_adapter | inline |
| base\_adapter() | stan::mcmc::base\_adapter | inline |
| disengage\_adaptation() | stan::mcmc::base\_adapter | inlinevirtual |
| engage\_adaptation() | stan::mcmc::base\_adapter | inlinevirtual |
| get\_stepsize\_adaptation() | stan::mcmc::stepsize\_var\_adapter | inline |
| get\_var\_adaptation() | stan::mcmc::stepsize\_var\_adapter | inline |
| set\_window\_params(unsigned int num\_warmup, unsigned int init\_buffer, unsigned int term\_buffer, unsigned int base\_window, std::ostream \*e=0) | stan::mcmc::stepsize\_var\_adapter | inline |
| stepsize\_adaptation\_ | stan::mcmc::stepsize\_var\_adapter | protected |
| stepsize\_var\_adapter(int n) | stan::mcmc::stepsize\_var\_adapter | inline |
| var\_adaptation\_ | stan::mcmc::stepsize\_var\_adapter | protected |


---

[ Stan Home Page ]

*© 2011–2014,
Stan Development Team.*
